# Burnout among early career psychiatrists in Russia – results of a cross-sectional study

**DOI:** 10.1192/j.eurpsy.2022.896

**Published:** 2022-09-01

**Authors:** E. Chumakov, A. Gvozdetsky, N. Petrova

**Affiliations:** 1 Saint-Petersburg University, Department Of Psychiatry And Addictions, Saint-Petersburg, Russian Federation; 2 St.-Petersburg Psychiatric Hospital No 1 named after P.P. Kaschenko, Day Inpatient Department, St Petersburg, Russian Federation; 3 North-Western State Medical University named after I.I. Mechnikov, Department Of Psychiatry And Addictions, St Petersburg, Russian Federation

**Keywords:** early career psychiatrists, burnout, ECPs

## Abstract

**Introduction:**

Despite the long history of burnout studies, the problem of burnout among psychiatric specialists in Russia is insufficiently studied. The risk of burnout is the highest among psychiatrists in the first 10 years of their career.

**Objectives:**

To assess the prevalence and severity of burnout in early career psychiatrists (ECPs) in Russia.

**Methods:**

An anonymous online survey of ECPs in Russia was conducted in July-August 2019 with a screening for burnout using the Maslach Burnout Inventory (MBI). The final sample consisted of 165 people (61.2% women; mean age 31.05±3.88). 95.2% of respondents lived in urban areas.

**Results:**

A high level of burnout according to at least one of the MBI scales was revealed in 71.5% of ECPs: in 79 (78.2%) women and 39 (60.9%) men (χ^2^(1)=5.74; p=0.017). Mean values of the MBI Emotional Exhaustion scale corresponded to 23.33±8.97 and 17.97±8.49 (U=1999.5; p=0.003), the MBI Depersonalization scale – 10.46±4.81 and 9.16±4.22 (U=2598.5; p=0.083), and the MBI Personal Accomplishment scale – 33.02±5.98 and 35.32±5.75 (U=2409.5; p=0.026) for women and men, respectively. The following risk factors for professional burnout were identified: female sex (OR=3.54 [95% CI: 1.96; 6.39], p<0.001), overlapping of several working positions (OR=2.44 [95% CI: 1.36; 4.37], p=0.003), difficulties in work due to changes in documentation requirements introduced since the start of career (OR=2.32 [95% CI: 1.31; 4.11], p=0.004).

**Conclusions:**

A high frequency of burnout among ECPs in Russia was revealed which suggests the urgent need for studies assessing the ways to prevent burnout in psychiatrists in Russia.

**Disclosure:**

No significant relationships.

